# Growth profiling, kinetics and substrate utilization of low-cost dairy waste for production of β-cryptoxanthin by *Kocuria marina* DAGII

**DOI:** 10.1098/rsos.172318

**Published:** 2018-07-11

**Authors:** Ruchira Mitra, Debjani Dutta

**Affiliations:** Department of Biotechnology, National Institute of Technology Durgapur, M.G. Avenue, Durgapur 713209, West Bengal, India

**Keywords:** β-cryptoxanthin, *Kocuria marina* DAGII, central composite design, artificial neural network, kinetics

## Abstract

The dairy industry produces enormous amount of cheese whey containing the major milk nutrients, but this remains unutilized all over the globe. The present study investigates the production of β-cryptoxanthin (β-CRX) by *Kocuria marina* DAGII using cheese whey as substrate. Response surface methodology (RSM) and an artificial neural network (ANN) approach were implemented to obtain the maximum β-CRX yield. Significant factors, i.e. yeast extract, peptone, cheese whey and initial pH, were the input variables in both the optimizing studies, and β-CRX yield and biomass were taken as output variables. The ANN topology of 4-9-2 was found to be optimum when trained with a feed-forward back-propagation algorithm. Experimental values of β-CRX yield (17.14 mg l^−1^) and biomass (5.35 g l^−1^) were compared and ANN predicted values (16.99 mg l^−1^ and 5.33 g l^−1^, respectively) were found to be more accurate compared with RSM predicted values (16.95 mg l^−1^ and 5.23 g l^−1^, respectively). Detailed kinetic analysis of cellular growth, substrate consumption and product formation revealed that growth inhibition took place at substrate concentrations higher than 12% (v/v) of cheese whey. The Han and Levenspiel model was the best fitted substrate inhibition model that described the cell growth in cheese whey with an *R*^2^ and MSE of 0.9982% and 0.00477%, respectively. The potential importance of this study lies in the development, optimization and modelling of a suitable cheese whey supplemented medium for increased β-CRX production.

## 1. Introduction

In recent years, microbial ability to grow on variable substrates has been widely explored for production of different secondary metabolites such as antibiotics, pigments and biosurfactants. The nutritional versatility of microbes makes them highly adaptable and culturable in laboratory conditions. This often leads to novel application of microbes on non-conventional substrates for industrial, pharmaceutical and environmental benefits. In our previous study, the inherent ability of *Kocuria marina* DAGII for production of β-cryptoxanthin (β-CRX) was reported [1]. β-CRX is a mono-oxygenated pro-vitamin A xanthophyll mostly provided by citrus fruits [2]. Administration of β-CRX has revealed various beneficial effects such as anti-oxidant, anti-inflammatory, anti-cancer, anti-obesity and anti-diabetic properties in different *in vitro* and *in vivo* studies [3,4]. β-CRX exerts cardio-protective action and also exhibits a unique regulatory role by maintaining bone health [5,6]. Research on health beneficiary roles of β-CRX is highly progressive, which has promoted development of innovative approaches for β-CRX production. Several methods such as extraction from citrus fruits, chemical transformation of commercial lutein to β-CRX as well as metabolic engineering of microbial hosts for β-CRX recovery have been developed [7]. However, to our best knowledge, no natural β-CRX producer has been reported to date except for *K. marina* DAGII [1]. As a result, *K. marina* DAGII holds high significance in research and development due to its ability to naturally produce β-CRX as the final and major product [8].

One of the major obstacles of carotenoid production, however, is the use of overpriced substrates [9]. Thus, replacement with a low-cost substrate might significantly help in cost reduction. The dairy and cheese industry is an integral part of India's economy and, by far, India is one of the largest producers and consumers of milk and dairy products. However, during production of cheese and other products such as cottage cheese, 85–95% of the milk volume is removed as whey [10]. Whey is the liquid remnant produced during separation of coagulated casein and fat from milk [11]. It is considered that whey consists of 55% milk nutrients of which the majority includes lactose (4.5–5% w/v), soluble proteins (0.6–0.8% w/v), lipids (0.4–0.5% w/v), mineral salts (8–10% of dried extract), lactic (0.05% w/v) and citric acids, non-protein nitrogen compounds (urea and uric acid) and B group vitamins [12]. Production of 1 kg of cheese results in the generation of approximately 9 kg of whey which is mostly disposed of unutilized [13]. It is roughly estimated that India accounts for an annual whey production of 2 million tonnes [14]. Thus, disposal of this huge quantity of whey is an environmental concern due to its high biological oxygen demand (30–50 g l^−1^) and chemical oxygen demand (60–80 g l^−1^) [15]. Mostly, whey is dumped into sewers or disposed of on land, which leads to detrimental impacts on health and the environment [16]. On the other hand, disposal into water bodies causes serious threat to aquatic life [10]. Thus, utilization of this whey for value addition could be a possible solution from health and environmental aspects. Cheese whey being a rich source of lactose could serve as an inexpensive fermentation medium for many microorganisms. To date, cheese whey has been used in different biotechnological processes for obtaining value added products such as ethanol, lactic acid, enzymes, biopolymers, biogas and single-cell protein [16]. In this context, our previous study on β-CRX production by *K. marina* DAGII using dual substrates has been extended by substituting the carbon sources with cheese whey for improved β-CRX production by *K. marina* DAGII [1].

Media design, optimization and kinetic modelling are crucial steps in a bioprocess. An effective production of the desired product requires development of a proper fermentation medium. Statistical approaches (response surface methodology (RSM) and artificial neural network (ANN)) are the ideal ways for media design and optimization of multivariable systems compared with the conventional ‘one-factor-at-a-time’ method which is not only tedious and time-consuming but also complicated for quantifying interactive effects of different factors in the process concerned [17]. In addition, analysis of rate equations for microbial growth, substrate uptake and product formation facilitates prediction of the behaviour of the biological system under different experimental conditions [18]. Thus establishment of mathematical models is also an indispensible step for commercial production of bioproducts.

In this present study, utilization of cheese whey as a substrate for β-CRX production was studied. The optimum medium composition for improved β-CRX production was validated using two statistical modelling approaches, RSM and ANN. Different unstructured kinetic models were developed to correlate between microbial growth, substrate concentration and product formation. The novelty of the research work lies in the utilization of a low-cost dairy residue, that is mostly disposed of as waste, for enhanced production of β-CRX. β-CRX production using cheese whey with optimization using ANN and validation with a kinetic model has not been reported so far.

## 2. Material and methods

### 2.1. Chemicals

All the media ingredients, i.e. brain heart infusion agar, yeast extract, peptone, glucose, maltose, sodium chloride, were purchased from Himedia, India. Solvents like methanol, petroleum benzene were HPLC grade and purchased from Merck, India. Reagents for lactose estimation, i.e. zinc acetate, phosphotungstic acid, glycine, sodium hydroxide, methylamine, sodium sulfite and lactose monohydrate, were purchased from Himedia, India. Standard β-CRX was obtained from Sigma-Aldrich, USA.

### 2.2. Microorganism, growth medium and inoculum preparation

*Kocuria marina* DAGII (accession number: KF498648) was isolated from soil in the Department of Biotechnology, NIT Durgapur, West Bengal, India [2]. The bacterium was maintained on a growth medium consisting of glucose (7.5 g l^−1^), maltose (10.0 g l^−1^), yeast extract (10.0 g l^−1^), peptone (5.0 g l^−1^) and sodium chloride (4.0 g l^−1^) with an initial pH of 7.9 [1]. Inoculum preparation was done as described by Mitra *et al.* [19]; 1% (v/v) of the inoculum with an optical density of 0.4–0.6 was used in all experiments.

### 2.3. Cheese whey preparation

Cheese whey was obtained from a local dairy shop (Durgapur, West Bengal, India). The precipitates were removed by centrifugation at 3220*g* for 10 min. The clear liquid was collected for further use.

### 2.4. Cultivation medium and culture conditions

Cheese whey was added to the growth medium and the concentration of the constituents was varied according to the experimental design. The Erlenmeyer flasks were incubated in a rotary shaker (150 r.p.m.) at 25°C for 120 h.

### 2.5. Dry weight, lactose measurement and carotenoid estimation

Biomass concentration was measured by the dry weight method and expressed in g l^−1^ [20]. The lactose content was determined by following the method of Nickerson *et al*. [21]. Briefly, cheese whey samples were reacted with methylamine in hot alkaline solution and the resulting red coloured solution was spectrophotometrically measured at 540 nm. The lactose content in the samples was determined from the standard curve of lactose and expressed in g l^−1^. β-CRX was extracted by a two-stage solvent extraction method as described by Mitra *et al*. [1]. Concentration of the extracted β-CRX was determined from the standard curve prepared using standard β-CRX and expressed as mg l^−1^ of culture.

### 2.6. Application of response surface methodology to optimize β-cryptoxanthin and biomass production by *Kocuria marina* DAGII

#### 2.6.1. Design of experiments

Design-Expert software (v. 8.0.7.1, Stat-Ease, Minneapolis, USA) was used for optimization of β-CRX and biomass production by *K. marina* DAGII. Optimization of the β-CRX and biomass production was done by central composite design (CCD). Four independent variables, namely yeast extract (A), peptone (B), cheese whey (C) and initial pH (D), were evaluated and coded to +1, 0 and −1 levels which corresponded to high, medium and low values, respectively (electronic supplementary material, table S1). In addition, the axial points were coded as +2 and −2 (electronic supplementary material, table S1). The four variables and their respective ranges were chosen based on the literature and preliminary experimental study. The β-CRX yield and biomass were modelled as the responses.

### 2.7. Artificial neural networks modelling

Artificial neural networks (ANNs) are powerful learning systems that are based on the principles of the human nervous system [22]. ANN is also known as neural nets, artificial neural system, parallel distributed processing system and connectionist system [23]. During the past few decades, ANN has emerged as an attractive tool for nonlinear multivariate modelling. In this study, a three-layer feed-forward network with sigmoid hidden neurons and linear output neurons was used to build an ANN model where the four variables (i.e. yeast extract, peptone, cheese whey and pH) served as input and the β-CRX yield and biomass were output. Since the data distribution in CCD experimental design is statistically uniform in the input domain, it is effectively used in ANN [24]. However for better accuracy, a higher number dataset is suggested, and thus 200 data points were additionally developed using the quadratic equation for β-CRX yield and biomass (electronic supplementary material, equations S1 and S2) [25]. In total, 230 data points were fed to the ANN architecture. The Levenberg–Marquardt back-propagation algorithm was employed for training the network. The ANN modelling was executed using MATLAB R2014a (v. 8.3, MathWorks®, USA).

### 2.8. Kinetic modelling

In this study, unstructured mathematical models for kinetic analysis were developed by taking into consideration the following assumptions:

(1) There was no oxygen limitation in the culture.
(2) There was no limitation by nitrogen.

#### 2.8.1. Microbial growth

A logistic kinetic model was used to simulate the growth of *K. marina* DAGII under varying cheese whey concentrations. Logistic equations are sets of equations that characterize growth in terms of maximum attainable biomass concentration, which is identical to the ecological concept of carrying capacity [26]. It is an independent model that can adequately describe the growth inhibition, a phenomenon that frequently occurs in batch culture [27]. The logistic model can be represented by equation (2.1), where *μ* is the specific growth rate (h^−1^), *X* is the biomass concentration (g l^−1^), *X*_m_ is the maximum biomass concentration (g l^−1^) that can be obtained from a particular fermentation system (corresponding to the carrying capacity) and (1 − (*X*/*X*_m_)) represents the unused carrying capacity [26].

2.1 $$\frac{\text{d}X}{\text{d}t}=\mu X\left(1-\frac{X}{X_{m}}\right).$$

Integration of the above equation using boundary condition as *X*(0) = *X*_0_ results in a sigmoidal curve representing both the exponential and stationary phases by the variation of *X* as a function of time, *t*.

2.2 $$X=\frac{X_{0}{\text{e}}^{\mu t}}{1-(X_{0}/X_{m})(1-{\text{e}}^{\mu t})}.$$

#### 2.8.2. Lactose consumption kinetics

Cheese whey was used as the substrate for growth and β-CRX production by *K. marina* DAGII. The lactose content in cheese whey was determined spectrophotometrically using the method of Nickerson *et al*. [21]. Further, the lactose utilization was modelled by logistic mass balance equation, which can be represented by equation (2.3), where *S*_L_ is lactose concentration (g l^−1^), $Y_{X/S_{L}}$ (g_cell biomass_ g_lactose_^−1^) is the maximum yield coefficient and *m*_C_ (g_lactose_ g_cell biomass_^−1^ h^−1^) is the maintenance coefficient [28].

2.3 $$-\frac{\text{d}S_{L}}{\text{d}t}=\frac{1}{Y_{X/S_{L}}}\frac{\text{d}X}{\text{d}t}+m_{C}X.$$

Integration of the equation (2.3) using boundary condition as *S*_L_(0) = *S*_L0_ at *t* = 0 results in equation (2.4), where *S*_L0_ is the initial lactose concentration.

2.4 $$-[S_{L}-S_{L0}]=\frac{1}{Y_{X/S_{L}}}{\left[X\right]}_{0}^{t}+m_{C}\int_{0}^{t}X\;\text{d}t.$$

Taking into consideration the equation for *X* (equation (2.2)), equation (2.4) can be represented as:

2.5 $$-[S_{L}-S_{L0}]=\frac{1}{Y_{X/S_{L}}}\left[\frac{X_{0}X_{m}{\text{e}}^{\mu t}}{X_{m}-X_{0}+X_{0}{\text{e}}^{\mu t}}-\frac{X_{0}X_{m}{\text{e}}^{0}}{X_{m}-X_{0}+X_{0}{\text{e}}^{0}}\right]+m_{C}\int_{0}^{t}\frac{X_{0}X_{m}{\text{e}}^{\mu t}}{X_{m}-X_{0}+X_{0}{\text{e}}^{\mu t}}\text{d}t.$$

To solve the integral part, temperature *T* was defined as an exponential function of *μt* [28] which can further be differentiated to

2.6 $$\text{d}T=\mu {\text{e}}^{\mu t}\text{d}t.$$

Hence, the equation (2.5) can be finally written as:

2.7 $$S_{L}=S_{L0}-\frac{X_{0}X_{m}{\text{e}}^{\mu t}}{Y_{X/S_{L}}(X_{m}-X_{0}+X_{0}{\text{e}}^{\mu t})}+\frac{X_{0}}{Y_{X/S_{L}}}-\frac{X_{m}m_{C}}{\mu }\text{ln}\frac{X_{m}-X_{0}+X_{0}{\text{e}}^{\mu t}}{X_{m}}.$$

The values of $Y_{X/S_{L}}$ and *m*_C_ were estimated from the nonlinear regression of *S*_L_ and *t*.

#### 2.8.3. Product formation kinetics

The β-CRX production by *K. marina* DAGII was described using the Leudeking–Piret kinetics equation [29]. According to this equation, the rate of product formation is directly and linearly proportional to growth rate and instantaneous biomass concentration and can be mathematically represented as:

2.8 $$\frac{\text{d}P}{\text{d}t}=\alpha \frac{\text{d}X}{\text{d}t}+\beta X,$$

where *α* is the growth associated product formation coefficient (exponential phase) and *β* is the non-growth associated product formation coefficient (stationary phase). The integrated form can be represented as:

2.9 $$\int_{0}^{P}\text{d}P=\alpha \int_{0-\Delta t}^{t-\Delta t}\frac{\text{d}X}{\text{d}t}\text{d}t+\beta \int_{0-\Delta t}^{t-\Delta t}X\text{d}t.$$

The term ‘Δ*t*’ was introduced to describe the delay in β-CRX production with respect to cell growth [28]. By substituting equation (2.2), equation (2.10) was generated which represents the nonlinear relationship between the product (*P*) and time (*t*).

2.10 $$\begin{array}{rl} P & =\alpha \left(\frac{X_{0}X_{m}{\text{e}}^{\mu (t-\Delta t)}}{X_{m}-X_{0}+X_{0}{\text{e}}^{\mu (t-\Delta t)}}-\frac{X_{0}X_{m}{\text{e}}^{-\mu \Delta t}}{X_{m}-X_{0}+X_{0}{\text{e}}^{-\mu \Delta t}}\right)\\ & \;+\frac{X_{m}\beta }{\mu }\left[\ln\left(\frac{X_{m}-X_{0}+X_{0}{\text{e}}^{\mu (t-\Delta t)}}{X_{m}}\right)-\ln\left(\frac{X_{m}-X_{0}+X_{0}{\text{e}}^{-\mu \Delta t}}{X_{m}}\right)\right]. \end{array}$$

### 2.9. Statistical analysis

All experiments were conducted in triplicate and results were reported as their averaged values. GraphPad Prism® v. 6.07 (GraphPad Software, Inc., USA) was employed in order to estimate the kinetic parameters from the model equations. The method of least squares was used to minimize the sum of the squares of the vertical distances between the points and the curve during regression analysis. The sum of the squares (SS) was calculated by equation (2.11), where *y_i_* and *f_i_* are the predicted data and experimental data, respectively, and *n* represents the length of the actual data period.

2.11 $$\text{SS}=\overset{n}{\underset{i=1}{\sum }}{\left(y_{i}-f_{i}\right)}^{2}.$$

The goodness of estimation was expressed by correlation coefficient *R*^2^, variance (*σ*), standard deviation of residuals (*S_y_*_.*x*_) and mean squared error (MSE). *R*^2^ and variance (*σ*) were determined using MS Excel. *S_y_*_.*x*_ (expressed in the same units as *y*-axis) was calculated from the sum of squares (SS) and degrees of freedom (d.f., equal to number of data points minus the number of parameters fit) as:

2.12 $$S_{y.x}=\sqrt{\frac{\text{SS}}{\text{d.f}.}}.$$

The MSE value was calculated by dividing the sum of squares by length of actual data period as:

2.13 $$\text{MSE}(\%)=\left(\frac{\text{SS}}{n}\right)\times 100.$$

## 3. Results and discussion

### 3.1. Screening of significant factors

The growth medium was supplemented with cheese whey and the effect of each medim component on β-CRX production was studied by deleting one or more factors. A set of 33 experiments with different medium composition was designed. Fourfold increase in β-CRX production (*p* < 0.05) was observed when glucose and maltose were substituted with cheese whey. Thus, yeast extract, peptone and cheese whey were selected as the significant contributing factors for CCD design. Additionally, pH was considered because it is an important parameter determining the growth of microbes.

The optimization of the factors responsible for β-CRX and biomass production by *K. marina* DAGII was performed.

#### 3.1.1. Experimental results and analysis of variance

**Figure 1.**
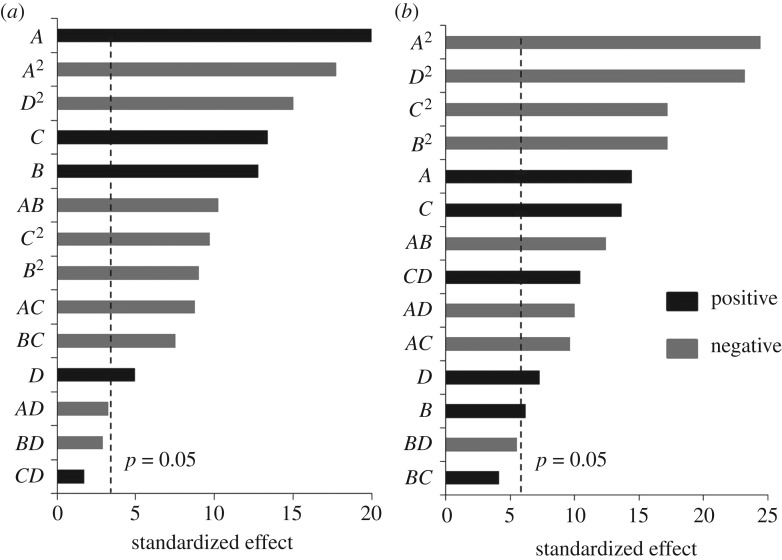
Standardized Pareto chart for β-CRX (*a*) and biomass (*b*).

**Figure 2.**
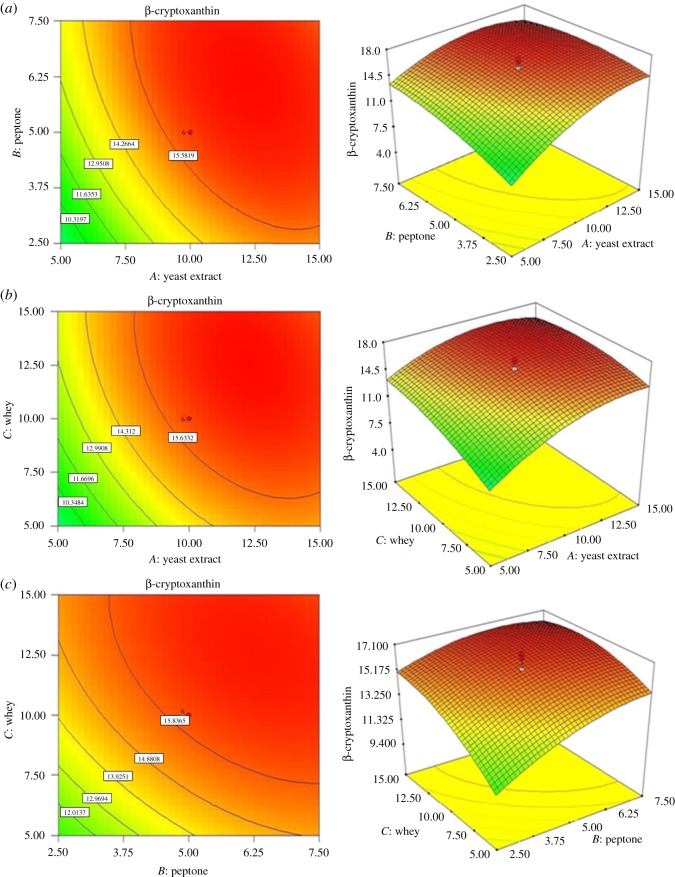
Contour plots and their corresponding 3D diagrams for β-CRX yield.

To examine the relationship between the responses and the four independent factors, a series of experiments were performed. The number of experiment required for the development of CCD was defined as:

3.1 $$N=2^{n}+2n+n_{C}.$$

where *N* is the total number of experiments, *n* is the number of factors and *n*_C_ is the number of central points [30]. With four factors, CCD consists of 16 factorial design runs, eight axial runs and six central points. The series of experiments and the corresponding values for the responses are shown in table 1. A multiple regression analysis was performed using Design-Expert and a quadratic model was suggested for the best fit model of β-CRX (*R*^2^ = 0.9888, ${R_{\mathrm{Adj}}}^{2}=0.9783$, ${R_{\mathrm{Pre}}}^{2}=0.9590$, Adeq. precision = 34.403) and biomass production (*R*^2^ = 0.9820, ${R_{\mathrm{Adj}}}^{2}=0.9801$, ${R_{\mathrm{Pre}}}^{2}=0.9606$, Adeq. precision = 37.344). Higher *R*^2^ values and Adeq. precision greater than 4 indicate high adequacy of a model. Thus, in our case, the values justified the model fitting. The results were analysed using analysis of variance (ANOVA) (table 2). The model *F*-values, *p*-values and lack of fit were used as a tool to evaluate the significance of the models. The *F*-value of 129.0 and 343.77 for β-CRX and biomass production, respectively, implied the model was significant. Model *p*-values (Prob > *F*) were significant (<0.0001) whereas the lack of fit was found to be insignificant (*p*-value_β-CRX_ = 0.8213, *p*-value_biomass_ = 0.2032). To understand the interacting effects, the *p*-values were further used to check the significance of the coefficients. A Pareto chart was designed to understand the contribution of each factor (figure 1). In the Pareto chart, effects have been standardized and arranged in the order of significance. The lengths of the bars are proportional to the magnitude of the estimated coefficients of the effects. The vertical line represents the minimum magnitude of the statistically significant effects of the response with a 95% CI. The coefficient estimates and the corresponding *p*-values suggested that individual factors were significant (*p* < 0.05) but yeast extract (*A*) had the largest effect, followed by cheese whey (figure 1). in the case of β-CRX production, the significance of peptone was more than that of pH. The interactive effect of yeast extract–peptone (*AB*) was found to be highest, followed by yeast extract–cheese whey (*AC*) and peptone–cheese whey (*BC*). The other interactive effects were found to be insignificant. All the quadratic terms (*A*^2^, *B*^2^, *C*^2^ and *D*^2^) were found to be significant (*p* < 0.0001). The final model equation in terms of coded factors is given below:

3.2 $$\begin{array}{rl} \beta \text{-CRX yield}(\text{mg }{\text{l}}^{-1}) & =16.09+2.17A+1.27B+1.34C+0.37D-0.98AB\\ & -0.81AC-0.18AD-0.67BC-0.14BD+(1.184\times 10^{-003})CD\\ & -1.84A^{2}-0.84B^{2}-0.92C^{2}-1.53D^{2}, \end{array}$$

where positive terms signified synergistic effect and negative terms signified antagonistic effect [31]. The contour diagram along with 3D response surface diagrams of the significant interactions are shown in figure. 2. Figure 2*a* shows the effect of yeast extract and peptone and their correlation between each other. When yeast extract and peptone concentrations were kept at minimum levels (i.e. 5 and 2.5 g l^−1^, respectively), the β-CRX yield was approximately 9 mg l^−1^. The β-CRX yield increased to 15 mg l^−1^ when the yeast extract concentration increased at fixed peptone concentration of 2.5 g l^−1^. However, when the peptone concentration was increased by keeping yeast extract fixed at 5 g l^−1^, β-CRX concentration increased to 13 mg l^−1^. This showed that the contribution of yeast extract was more compared with peptone for β-CRX production and, thus, justified the variation in *F*-value. Probably the levels of amino acids and small peptides present in yeast extract were higher and they were easily transported and used by the cell for metabolite production [32]. Figure 2*b* shows an approximate increase in β-CRX yield from 9 to 13 mg l^−1^ when cheese whey concentration was increased keeping yeast extract concentration fixed at 5 g l^−1^. When both the factors were kept at their maximum levels, the β-CRX yield was almost 16 mg l^−1^. In figure 2*c*, it was observed that at a cheese whey concentration of 15% (v/v), the β-CRX yield increased from approximately 15 to 16.2 mg l^−1^ when peptone was varied from 2.5 to 7.5 g l^−1^. Even at low concentration of cheese whey (i.e. 5% v/v) and peptone (2.5 g l^−1^), the β-CRX yield was considerably higher (approx. 11 mg l^−1^). This suggested that the nutritional components of cheese whey enhanced the β-CRX production by *K. marina* DAGII.

**Table 1.** CCD design matrix of independent variables and their corresponding values of responses.

	factor 1	factor 2	factor 3	factor 4	response 1	response 2
run	*A*: yeast extract (g l^−1^)	*B*: peptone (g l^−1^)	*C*: cheese whey (% v/v)	*D*: initial pH (unit)	β-CRX yield (mg l^−1^)	biomass (g l^−1^)
1	10	10	10	7.75	14.8140	3.82
2	10	5	10	7.75	17.0450	5.16
3	15	7.5	15	7	13.0575	3.69
4	15	7.5	5	7	14.0800	3.75
5	15	2.5	15	8.5	13.9652	4.07
6	5	2.5	5	8.5	04.2459	2.67
7	5	2.5	5	7	03.3500	2.43
8	10	5	10	7.75	16.7555	5.31
9	5	7.5	15	8.5	12.8590	4.15
10	20	5	10	7.75	13.0005	3.67
11	15	7.5	5	8.5	13.2780	3.23
12	5	2.5	15	7	08.6440	2.92
13	5	7.5	15	7	12.4416	3.66
14	15	2.5	5	7	11.0203	4.07
15	10	5	10	7.75	15.5215	5.21
16	10	0	10	7.75	10.2377	3.88
17	10	5	0	7.75	09.4441	3.45
18	10	5	10	9.25	10.6607	3.46
19	5	7.5	5	7	09.1972	3.15
20	10	5	10	7.75	15.9500	5.14
21	5	2.5	15	8.5	10.2473	3.92
22	10	5	10	7.75	15.6593	5.12
23	5	7.5	5	8.5	10.4346	3.17
24	0	5	10	7.75	04.1105	2.64
25	10	5	10	6.25	08.8854	3.05
26	15	2.5	5	8.5	12.4019	3.64
27	15	7.5	15	8.5	13.8017	3.90
28	10	5	20	7.75	15.0266	4.25
29	10	5	10	7.75	15.6155	5.12
30	15	2.5	15	7	13.9983	3.95

**Table 2.** Analysis of variance for the quadratic model for β-CRX and biomass.

source	sum of squares	degrees of freedom	mean square	*F*-value	*p* > *F*
β-CRX yield					
model	379.5	14	27.11	94.32	<0.0001
*A*-yeast extract	112.51	1	112.51	391.48	<0.0001
*B*-peptone	38.58	1	38.58	134.24	<0.0001
*C*-cheese whey	43.13	1	43.13	150.06	<0.0001
*D*-pH	3.37	1	3.37	11.73	0.0038
*AB*	15.24	1	15.24	53.02	<0.0001
*AC*	10.44	1	10.44	36.31	<0.0001
*AD*	0.51	1	0.51	1.78	0.2017
*BC*	7.11	1	7.11	24.74	0.0002
*BD*	0.32	1	0.32	1.1	0.3105
*CD*	2.24 × 10^−5^	1	2.24 × 10^−5^	7.81 × 10^−5^	0.9931
*A*^2^	92.39	1	92.39	321.48	<0.0001
* B*^2^	19.48	1	19.48	67.78	<0.0001
* C*^2^	22.98	1	22.98	79.97	<0.0001
* D*^2^	64.29	1	64.29	223.69	<0.0001
residual	4.31	15	0.287393		
lack of fit	2.2	10	0.220255	0.52	0.8213
pure error	2.11	5	0.421671		
cor total	383.81	29			
biomass					
model	18.98	14	1.355677	133.56	<0.0001
*A*-yeast extract	1.65	1	1.651913	162.75	<0.0001
*B*-peptone	0.034	1	0.033863	3.34	0.0477
*C*-cheese whey	1.36	1	1.363982	134.38	<0.0001
*D*-pH	0.16	1	0.158681	15.63	0.0013
*AB*	0.7	1	0.697851	68.75	<0.0001
*AC*	0.33	1	0.33394	32.9	<0.0001
*AD*	0.35	1	0.351501	34.63	<0.0001
*BC*	6.20 × 10^−5^	1	6.2 × 10^−5^	6.1 × 10^−3^	0.9387
*BD*	0.033	1	0.03317	3.27	0.0907
*CD*	0.4	1	0.397373	39.15	<0.0001
*A*^2^	7.02	1	7.015331	691.17	<0.0001
* B*^2^	3.04	1	3.043525	299.86	<0.0001
* C*^2^	3.03	1	3.032115	298.73	<0.0001
*D*^2^	6.37	1	6.368598	627.45	<0.0001
residual	0.15	15	0.01015		
lack of fit	0.12	10	0.012373	2.17	0.2032
pure error	0.029	5	0.005704		
cor total	19.13	29			

In the case of biomass production, the interactive effects of yeast extract–peptone (*AB*), yeast extract–cheese whey (*AC*), yeast extract–pH (*AD*) and cheese whey–pH (*CD*) were found to be significant (figure 1). Similar to β-CRX production, all the quadratic terms were significant. The final equation for regression was found to be

3.3 $$\begin{array}{rl} \text{biomass} & =5.18+0.26A+0.038B+0.24C+0.081D\\ & \;-0.21AB-0.14AC-0.15AD+(1.969\times 10^{-003})BC-0.046BD\\ & \;+0.16CD-0.51A^{2}-0.33B^{2}-0.33C^{2}-0.48D^{2}. \end{array}$$

**Figure 3.**
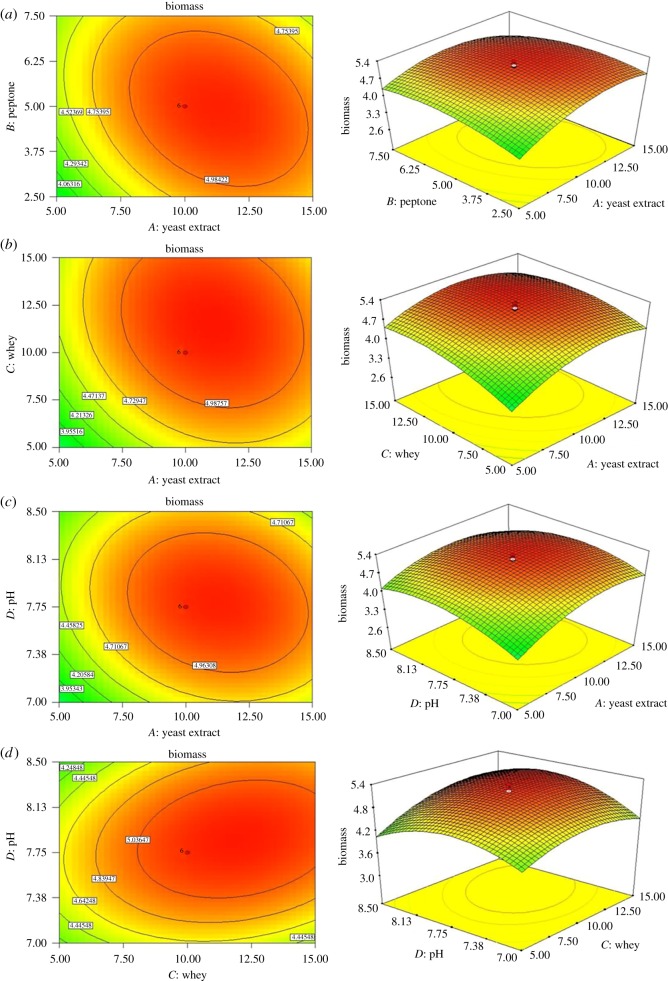
Contour plots and their corresponding 3D diagrams for biomass.

Figure 3 shows the contour plots and the corresponding 3D diagrams for biomass production. Figure 3*a* shows an increase in biomass yield from 3.76 to 4.7 g l^−1^ when the yeast extract was varied from 5.0 to 15 g l^−1^ at constant peptone concentration of 2.5 g l^−1^. Figure 3*b* depicted the relationship between yeast extract and cheese whey and it was clearly evident that biomass production gradually increased with cheese whey concentration. Figure 3*c*,*d* shows the relationship of yeast extract and cheese whey with pH, respectively. Both the figures suggested that variation of pH increased the biomass; however, above pH 7.75 the biomass reduced. Probably alkaline pH slowed the bacterial growth [33].

The electronic supplementary material, figure S1 shows the correlation between the experimental and predicted values of β-CRX and biomass yield obtained from the model, respectively. Distribution of the data points shows adequate agreement between the experimental and predicted values. This proved that the predicted quadratic model was appropriate to navigate the design space defined by the CCD. CCD has long been used as an important statistical tool for optimization. For instances in 2007, cell growth and carotenoid biosynthesis in *Xanthophyllomyces dendrorhous* was successfully optimized using CCD. Similarly, Imamoglu *et al*. [34] statistically evaluated the physical growth parameters of *Dunaliella salina* strain EgeMacc-024 during batch production of chlorophyll *a*. In 2015, hydrolysis of cassava fibrous waste (a hugely produced solid waste during processing of cassava tubers in sago industries in India) to obtain maximum glucose yield was done using CCD and a quadratic polynomial equation predicting the optimal points was developed [35]. In another study, CCD was employed for enhanced co-production of xylanase and lichenase by *Bacillus subtilis* D3d using different agro-industrial residues [36].

#### 3.1.2. Optimization and verification study

The optimum values of the four variables for β-CRX and biomass production were obtained by numerical optimization using Design-Expert 8.0.7.1. β-CRX yield and biomass were maximized by keeping the four independent factors ‘in range’. The maximum production was obtained at 11.47 g l^−1^ yeast extract, 5.29 g l^−1^ peptone, 12.00% (v/v) cheese whey and pH 7.83. The predicted values of β-CRX (16.95 mg l^−1^) and biomass (5.23 g l^−1^) yield were further verified by conducting three additional experiments at the obtained optimum condition. The average experimental values were found to be 17.14 mg l^−1^ and 5.35 g l^−1^, respectively, which were in good agreement with the predicted values and, thus, validated the model obtained by CCD. In a similar study by Khodaiyan *et al*. [37], maximum canthaxanthin yield of 2.871 ± 0.076 mg l^−1^ was obtained at whey lactose concentration of 55.54 g l^−1^, yeast extract concentration of 7.36 g l^−1^ and pH of 7.66.

### 3.2. Prediction of responses with artificial neural network

**Figure 4.**
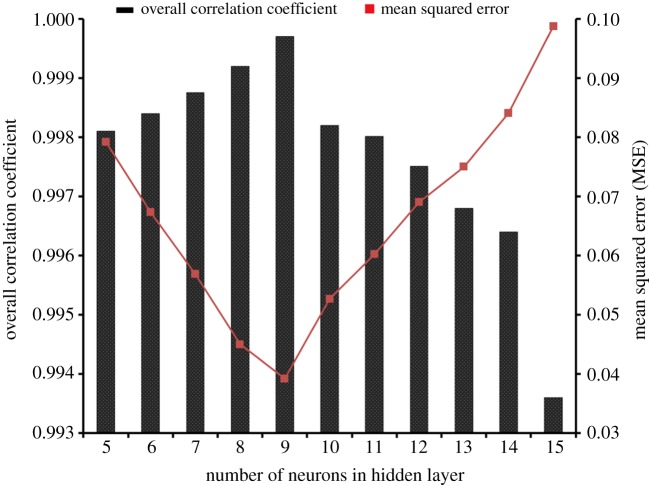
Effect of neuron number on ANN model.

The electronic supplementary material, figure S2 shows the ANN model constructed with input layer, hidden layer and output layer. The input layer consisted of four neurons, i.e. concentrations of yeast extract, peptone, cheese whey and pH. The output layer consisted of two neurons, i.e. β-CRX yield and biomass. In a feed-forward back-propagation algorithm, input information is transmitted to the output layer through neurons of a hidden layer. To determine the number of neurons in the hidden layer, MSE and *R*^2^ of different neural networks were evaluated on a trial and error basis (figure 4). A network consisting of a hidden layer with nine neurons gave the best result. The ANN model was trained with 160 samples, validated with 35 samples and the accuracy of the model and prediction were further tested with 35 samples.

**Figure 5.**
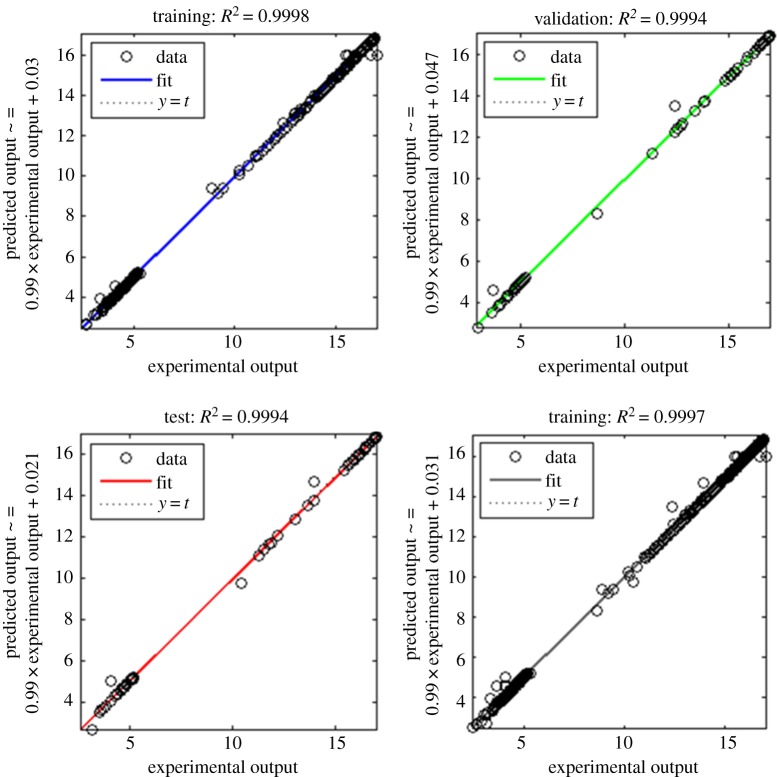
Experimental result versus ANN predicted output.

The Levenberg–Marquardt algorithm used in the present study is a standard iterative technique that localizes local minimum of a multivariate function by expressing sum of squares of several nonlinear, real-valued functions in a short time [38]. Using this algorithm training automatically stops when generalization stops improving, which is indicated by an increase in the MSE of the validation samples. From the electronic supplementary material, figure S3, it was observed that best validation was achieved at epoch 7. The correlations between the experimental and the predicted results are given in figure 5. As indicated by the *R*^2^ values, ANN served as a reliable prediction model in our study. Moreover, the MSE for the entire dataset was significantly low, as depicted by the error histogram (electronic supplementary material, figure S4), which suggested that the ANN model possessed good approximation characteristics. The ANN predicted yield of β-CRX and biomass was found to be 16.99 mg l^−1^ and 5.33 g l^−1^, respectively. It was evident from the values of *R*^2^ and predicted yield that even though both the models (RSM as well as ANN) fitted well to the experimental design, ANN offered better predictive and approximation accuracy. The better predictive accuracy of ANN can be attributed to the fact that it can universally approximate the nonlinearity of any system and additionally it has the ability to calculate multiple responses in a single run. By contrast, RSM can be implemented only upto second-order polynomial and it must be run multiple times for multiple responses (run number equal to number of responses to be predicted) [39]. In 2014, Rafigh *et al*. [40] successfully modelled the curdlan production from *Paenibacillus polymyxa* using RSM and ANN and suggested that ANN reported more stable responses. In a study by Azad *et al*. [41], the ANN model showed distinct superiority compared with the RSM model during optimization of process parameters for adsorption of ternary dyes by nickel doped ferric oxyhydroxide FeO(OH) nanowires on activated carbon. Recently, a feed-forward back-propagation algorithm was effectively implemented to develop an ANN model for improved *ϵ*-polylysine production by the marine bacterium *B. licheniformis* [25].

From this entire work, it was found that substitution of the previous carbon source (i.e. glucose and maltose) with the help of cheese whey gave fourfold increase in β-CRX production and 1.7-fold increase in biomass. Finally, validation of RSM statistical data with the help of the ANN method resulted into 4.67- and 2.34-fold increase in β-CRX yield and biomass, respectively. Probably cheese whey utilization increases the acetyl-CoA pool in the bacterium which eventually increases the production of isopentenyl pyrophosphate (IPP) and dimethylallyl diphosphate (DMADP) through the mevalonic acid pathway. As IPP and DMADP are the key precursors of carotenogenesis, an increased concentration of both the precursors is leading to increased β-CRX production by *K. marina* DAGII.

### 3.3. Kinetic modelling of *Kocuria marina* DAGII

Kinetic modelling helps in accessing the interaction between microbial growth and the surrounding environment. It helps in predicting the behaviour of microbial processes under different environmental conditions. Most importantly, the quantitative knowledge of kinetic parameters helps in analysis, optimization, design and operation of biological processes at large scale conditions [42]. Thus, development of a mathematical model is an important step for better understanding of microbial kinetics. Structured mathematical models involve intracellular metabolism of the biosystem, which makes the process complicated. On the other hand, unstructured models consider only biomass as its principal variable which makes its implementation simple and robust [43].

#### 3.3.1. Microbial growth kinetics

**Figure 6.**
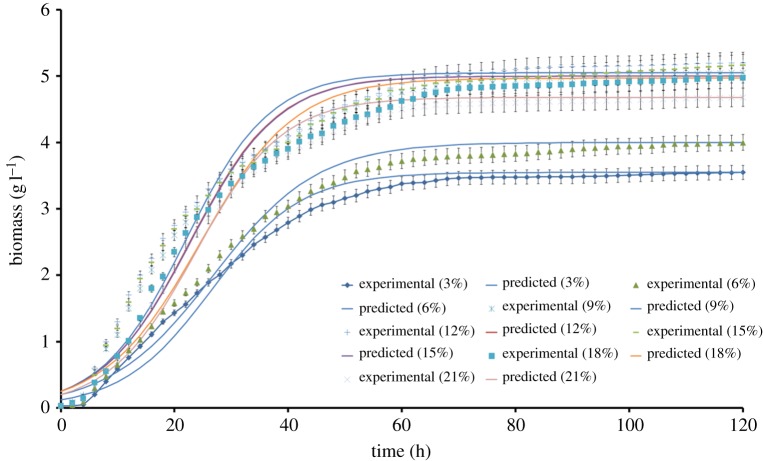
Fitting of the experimental data to the logistic model describing cell growth over time at 3.0–21.0% (v/v) of initial cheese whey concentration.

*Kocuria marina* DAGII showed a classical growth trend in the presence of cheese whey as substrate. Irrespective of the cheese whey concentration, the *K. marina* DAGII cells entered the exponential phase after a lag phase of 4 h. During lag phase, the physiologically active bacterial cells adapted to the new experimental environment but no apparent growth was observed. Once the acclimatized cells entered the exponential phase the biomass increased at a constant rate; however, the growth slowed down during its transition to stationary phase at 38–48 h. In our previous study, it was observed that in the presence of glucose and maltose as carbon source, *K. marina* DAGII achieved stationary phase at 25 h [1]. However, substitution of the carbon source with cheese whey delayed the onset of nutrient depletion by 24 h and, thus, the exponential phase continued for a longer time period. A logistic equation was employed to describe the growth kinetics of the *K. marina* DAGII and it was observed that the simulated growth profile was in good agreement with the experimental growth curve for all the cheese whey concentrations, as indicated in figure 6. The kinetic parameters were obtained by fitting the experimental data into the logistic equations (table 3). The low values of statistical parameters such as sum of squares (SS), standard deviation of residuals (*S_y.x_*) and MSE signified the good fit of the model. Additionally, the high *R*^2^ values (>0.90) suggested that the logistic model successfully described the sigmoidal growth pattern of the bacterium taking into consideration the growth inhibition that took place in the stationary phase. According to a number of researchers, logistic equations have been successful in predicting sigmoidal growth patterns of different bacteria under a batch mode of operations [44]. However, it was noteworthy that the specific growth rate (*μ*) and maximum biomass yield (*X*_m_) of *K. marina* DAGII increased when the cheese whey concentration was increased from 3 to 12% (v/v), but after 12% (v/v) a gradual decrease was noted (table 3). This indicated that above 12% (v/v), cheese whey played an inhibitory role during the growth of *K. marina* DAGII and thus necessitated the implementation of a substrate inhibition model for estimation of inhibition parameters. Cell growth during batch production of lutein by heterotrophic *Chlorella* decreased as the glucose concentration was increased from 10 to 60 g l^−1^ [45]. Similarly, carotenoid production by *X. dendrorhous* using *Yucca fillifera* date juice as substrate was studied by Luna-Flores *et al*. [46], and it was found that cell growth decreased when substrate concentration was increased from 20 to 40 g l^−1^. In 2016, Kim *et al*. [47] reported that specific growth rate of *Klebsiella oxytoca* during 2,3-butanediol production increased with increase in glucose concentration up to 32 g l^−1^ and thereafter gradually decreased.

**Table 3.** Kinetic parameter for growth at different cheese whey concentrations.

parameter estimation	cheese whey concentration (% v/v)						
microbial growth	3	6	9	12	15	18	21
*X*_0_ (g l^−1^)	0.0300	0.0320	0.0290	0.0310	0.0350	0.0330	0.0260
*X*_m_ (g l^−1^)	3.5500	4.0000	5.0500	5.2000	5.1700	4.9700	4.6800
*μ*(*h*^−1^)	0.0421	0.0837	0.1243	0.1518	0.1427	0.1260	0.1074
*R*^2^	0.9805	0.9827	0.9733	0.9681	0.9595	0.9715	0.9859
*σ*	1.4351	1.7424	2.5258	2.5442	2.5215	2.5854	2.3475
SS	1.801	1.599	1.968	2.043	2.1631	1.983	1.472
*S_y.x_* (g l^−1^)	0.1718	0.1619	0.1796	0.1830	0.1883	0.1803	0.1553
MSE (%)	2.9524	2.6213	3.2262	3.3491	3.5460	3.2508	2.4131
lactose consumption							
*S*_L0_(g l^−1^)	3.5	3.91	4.06	4.22	4.36	4.58	4.76
*m*_c_ (g_lactose_ g_biomass_^−1^ h^−1^)	0.0027	0.0029	0.0031	0.0034	0.0029	0.0029	0.0028
$Y_{X/S_{L}}$ (g_biomass_ g_lactose_^−1^)	12.28	13.22	14.82	16.95	14.9	13.31	12.25
* R*^2^	0.9857	0.9832	0.9948	0.9989	0.9889	0.9809	0.9913
* σ*	0.2809	0.2975	0.2187	0.2032	0.2634	0.2991	0.2491
SS	0.0387	0.0486	0.0181	0.0046	0.0456	0.0847	0.0376
* S_y.x_* (g l^−1^)	0.0656	0.0735	0.0449	0.0226	0.0712	0.0970	0.0646
MSE (%)	0.3525	0.4420	0.1649	0.0420	0.4149	0.7705	0.3419
β-CRX production							
* α* (mg_β-CRX_ g_biomass_^−1^)	1.029	1.294	1.598	1.958	1.607	1.548	1.498
* β* (mg_β-CRX_ g_biomass_^−1^ h^−1^)	0.0150	0.0174	0.0198	0.0199	0.0199	0.0192	0.01813
* *Δ*t* (h)	2.546	2.582	2.494	2.161	2.055	2.347	2.161
* R*^2^	0.9991	0.9987	0.9998	0.9993	0.9989	0.9992	0.9992
* σ*	0.2230	0.2360	0.2085	0.2203	0.2263	0.2318	0.2338
SS	0.5919	0.6499	0.6018	0.6113	0.6284	0.646	0.5968
* S_y.x_* (mg l^−1^)	0.0739	0.0812	0.0752	0.0764	0.0785	0.0807	0.0746
MSE (%)	0.0538	0.0590	0.0547	0.0555	0.0571	0.0587	0.0542

#### 3.3.2. Substrate consumption

**Figure 7.**
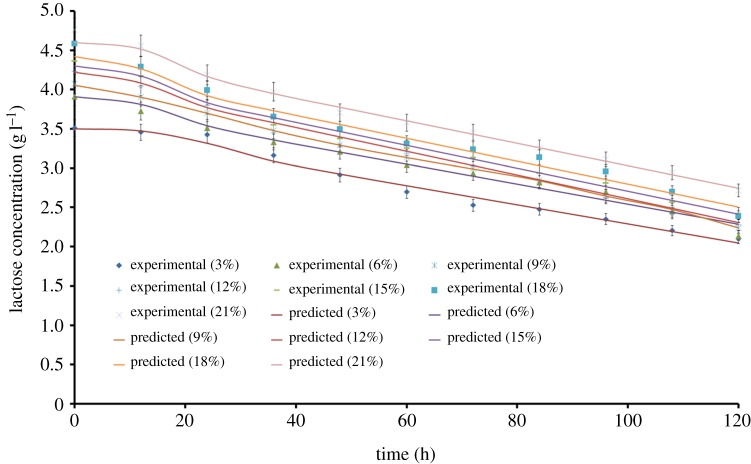
Fitting of the experimental data to the model describing lactose consumption with time at varying cheese whey concentrations.

Substrate utilization is an important factor governing cell growth and β-CRX production. The lactose utilization results were incorporated into the logistic mass balance equation and the kinetic parameters were calculated as shown in table 3. The *S*_0_, $Y_{X/S_{L}}$ and *m*_C_ at the varying cheese whey concentrations were calculated. The values of *R*^2^, SS, *Sy.x*, MSE% depicted that the fitting results were satisfactory (table 3). Moreover, the graphical representation of the experimental and predicted lactose consumption clearly justified the fitness of the experimental data into the logistic mass balance model (figure 7). The values of $Y_{X/S_{L}}$ exhibited an increasing trend when the cheese whey concentration was increased from 3 to 12% (v/v). After 12% (v/v), the $Y_{X/S_{L}}$ decreased gradually. It could be inferred that after a certain concentration of lactose, the bacterium was unable to use the excess substrate. In a similar study by Goswami *et al*. [48], it was reported that the yield coefficient decreased when the glucose concentration was increased beyond 15 g l^−1^ during batch production of canthaxanthin by *Dietzia maris* NITD. The maintenance coefficient (*m*_C_) varied slightly with cheese whey concentration. According to Shuler & Kargi [26], the maintenance coefficient (*m*_C_) is the parameter that describes the specific rate of substrate uptake for cellular maintenance and can be represented as

3.4 $$m_{C}=\frac{\text{d}S/\text{d}t_{m_{C}}}{X}.$$

The maintenance coefficient is considered as a measure for substrate utilization for non-growth related activities such as energy required by the cell for repairing damaged cellular components, transferring nutrients and products in and out the of cell, for mobility and for adjusting osmolarity of the cell interior. Thus, it is likely that yield coefficient values will depend on the maintenance coefficient. Depending upon the environmental conditions of the cell, the values of *m*_C_ might vary from 0.02 to 4.0 g_lactose_ g_biomass_^−1^ h^−1^ [49]. During the kinetic modelling study of hyaluronic acid production by *Streptococcus zooepidemicus*, ‘*m*_C_’ ranged from 0.04 to 1.56 (g_glucose_ g_cell biomass_^−1^ h^−1^) with variation in glucose concentration [28]. The ‘*m*_C_’ value was found to be 0.0699 g g^−1^ d^−1^ during ajmalicine production from hairy roots of *Catharanthus roseus* using sucrose as the substrate [50]. Thus, the above discussion suggests that the logistic mass balance equations adequately describe the lactose consumption by *K. marina* DAGII during β-CRX production.

#### 3.3.3. β-Cryptoxanthin production

**Figure 8.**
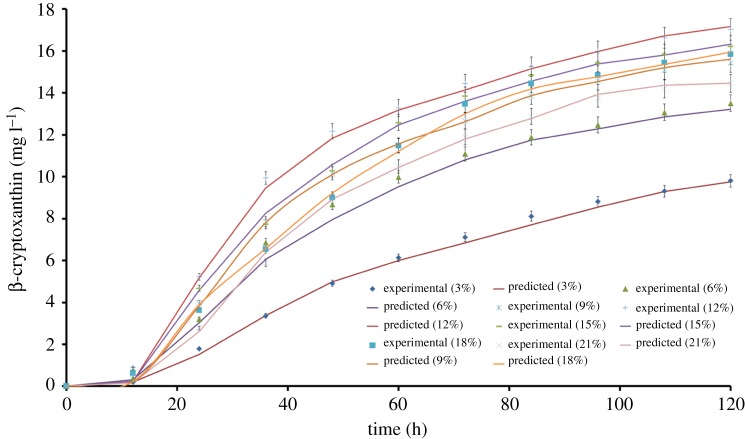
Fitting of the experimental data to the Luedeking–Piret model describing β-CRX production over time at 3.0–21.0% (v/v) of initial cheese whey concentrations.

During the fermentation, β-CRX production increased with time and the maximum concentration was obtained at 120 h of incubation. The corresponding β-CRX concentration at 3, 6, 9, 12, 15, 18 and 21% (v/v) of cheese whey was found to be 9.8, 13.5, 15.4, 17.14, 16.23, 15.84, 14.45 mg l^−1^, respectively. The experimental data were fitted to the Luedeking–Piret model and the values of *α*, *β* and *Δt* were determined from the resulting nonlinear regression (table 3). The high *R*^2^ values indicated that the experimental data fitted well into the model. The observation was further justified by the low values of SS, *Sy.x* and MSE%. Moreover, the predicted data was in accordance with the experimental data as depicted in figure 8. The variation in *α* and *β* values suggested that the maximum yield was obtained at 12% (v/v) of cheese whey. Cheese whey concentrations beyond 12% (v/v) inhibited the β-CRX production (table 3). The decrease could be due to catabolic repression and reduced specific growth rate at higher concentrations of cheese whey [47]. During the fermentation process, the cells serve as the factory for metabolite production, probably; thus growth inhibition at higher substrate concentration results in the reduced product formation. The *Δt* was found to be in the range of 2.0–2.6 h which indicated that the pigment production was mostly growth dependent and also justified the higher magnitude of *α* compared to *β*. In the study by Don & Shoparwe [28], production kinetics showed that lag time between the hyaluronic acid production and cell growth of *S. zooepidemicus* varied from 0.49 to 2.16 h. The Luedeking–Piret model was also successfully implemented by Gutiérrez-Arnillas *et al*. [51] to elucidate the metabolic characteristics of lipolytic enzymes synthesized by halophilic microorganisms. The study further reported the product to be a secondary metabolite based on the values of *α* and *β*.

#### 3.3.4. Model development for substrate inhibition

Based on the results of previous sections, it was observed that higher concentrations of cheese whey inhibited the cell growth of *K. marina* DAGII. It was even plausible that higher product concentration was responsible for the decreased cell growth. However, in the present study, the specific growth rate remained fairly constant with respect to time during the exponential phase of the bacterial growth which indicated that the inhibition occurred due to the substrate.

**Figure 9.**
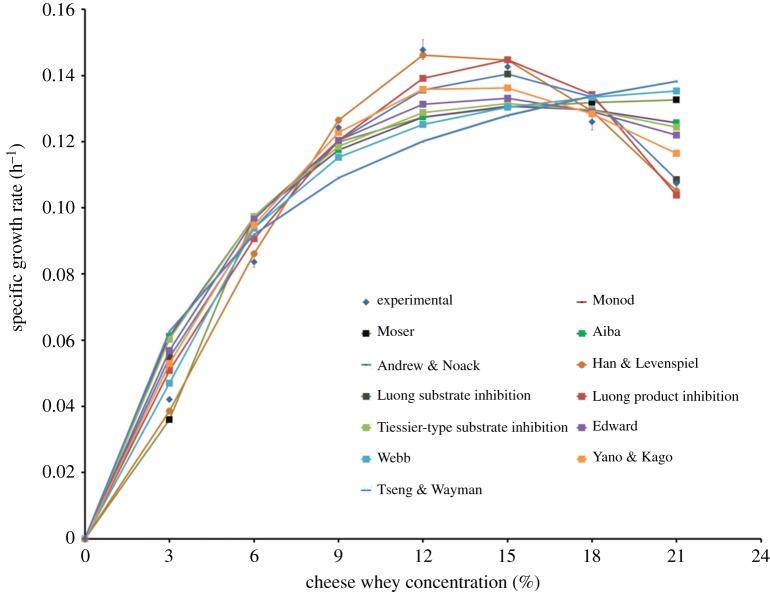
Comparison of the experimental data and model simulations at different initial cheese whey concentrations.

When the *μ* values (obtained from the logistic model) were fitted to the Monod model, it failed to validate the experimental data. Thus, other unstructured kinetic models were used and the data were validated (electronic supplementary material, table S2). The *μ* values at all the varying cheese whey concentrations were fitted to the models and the biokinetic parameters such as *μ*_max_ (maximum specific growth rate), *K*_s_ (Monod half saturation constant), *K_i_* (substrate inhibition concentration), *S*_m_ (maximum substrate inhibition constant above which cells cease to grow), and *K*_1_, *K*_2_, *n* and *m* (constants correlating between *μ* and substrate) were evaluated and the results shown in the electronic supplementary material, table S3. The graphical representation of the simulated and the experimental data is shown in figure 9. The fitness of the models was quantified by evaluating *R*^2^, *σ*, SS, *Sy.x* and MSE%. Based on these statistical analyses, it was observed that the highest *R*^2^ value and lowest *σ*, SS, *Sy.x* and MSE% values were obtained in the case of the Han and Levenspiel model. The Han and Levenspiel model is a generalized form of Monod kinetics which is based on the assumption that there exists a critical substrate concentration above which cells cease to grow, and the constants of the Monod equation are functions of this limiting inhibitor concentration [52]. The model equation describing the cell growth with cheese whey as substrate can be represented as given in equation (3.5), where *S* is the initial cheese whey concentration:

3.5 $$\mu =0.2385{\left(1-\frac{S}{27.40}\right)}^{0.5576}\left(\frac{S}{S+22.06{\left(1-(S/27.40)\right)}^{3.868}}\right).$$

## 4. Conclusion

Optimization and kinetic modelling of a fermentation process have been successfully described in the present study. The statistical tools RSM and ANN were used for elucidating the optimal condition for β-CRX production by *K. marina* DAGII using cheese whey as the substrate. Significantly high β-CRX yield was achieved when carbon sources were substituted with 12% (v/v) cheese whey. The results suggested that both RSM and ANN showed stable predictive responses but ANN was more accurate for data fitting and estimation capabilities. The kinetic models for cell growth, substrate consumption and product formation were analysed. Logistic equations adequately described the growth profile of *K. marina* DAGII under varying cheese whey concentrations and the substrate consumption was well defined by logistic mass balance equation. The product formation coefficients were evaluated using the Luedeking–Piret model with high accuracy. With reference to the substrate inhibition the Han and Levenspiel model fitted best with the experimental data. In our opinion, utilization of cheese whey for production of valuable products such as carotenoids is an optimistic approach for value addition and a cleaner environment.

## Supplementary Material

Supplementary material

## Data accessibility

Our data are deposited at Dyrad (http://dx.doi.org/10.5061/dryad.jd8bh) [53].
