# Supplementary material for: Growth profiling, kinetics and substrate utilization of low-cost dairy waste for production of β-cryptoxanthin by Kocuria marina DAGII
Source: R Soc Open Sci. 2018 Jul 11;5(7):172318. doi: 10.1098/rsos.172318 (PMC6083662; doi:10.1098/rsos.172318)
Supplement: Supplementary material [file rsos172318supp1.pdf]

Supplementary files

Regression equation for β-CRX yield and biomass

β-CRX yield =-191.55404 +2.98452*Yeast Extract +3.75104*Peptone +1.58765*Whey +43.53464*pH -0.078069* Yeast Extract * Peptone -0.032304*Yeast Extract * Whey -0.047721*Yeast Extract * pH - 0.053334*Peptone*Whey -0.075028*Peptone*pH +3.15833E-004*Whey*pH -0.073413* Yeast $Extract^2$ -0.13484* $Peptone^2$ -0.036614* $Whey^2$ -2.72166*pH^2 **(Eq. S1)**

Biomass = -55.02614 +0.90470 * Yeast Extract +0.90170 * Peptone +0.044973 * Whey +13.48274 * pH -0.016707 * Yeast Extract * Peptone -5.77875E-003 * Yeast Extract * Whey -0.039525 * Yeast Extract * pH +1.57500E-004 * Peptone * Whey -0.024283 * Peptone * pH +0.042025 * Whey * pH -0.020229 * Yeast $Extract^2$ -0.053298 * $Peptone^2$ -0.013299 * $Whey^2$ -0.85664 * pH^2 **(Eq. S2)**

Fig. S1 Predicted vs. Actual yields for β-CRX (A) and biomass (B)

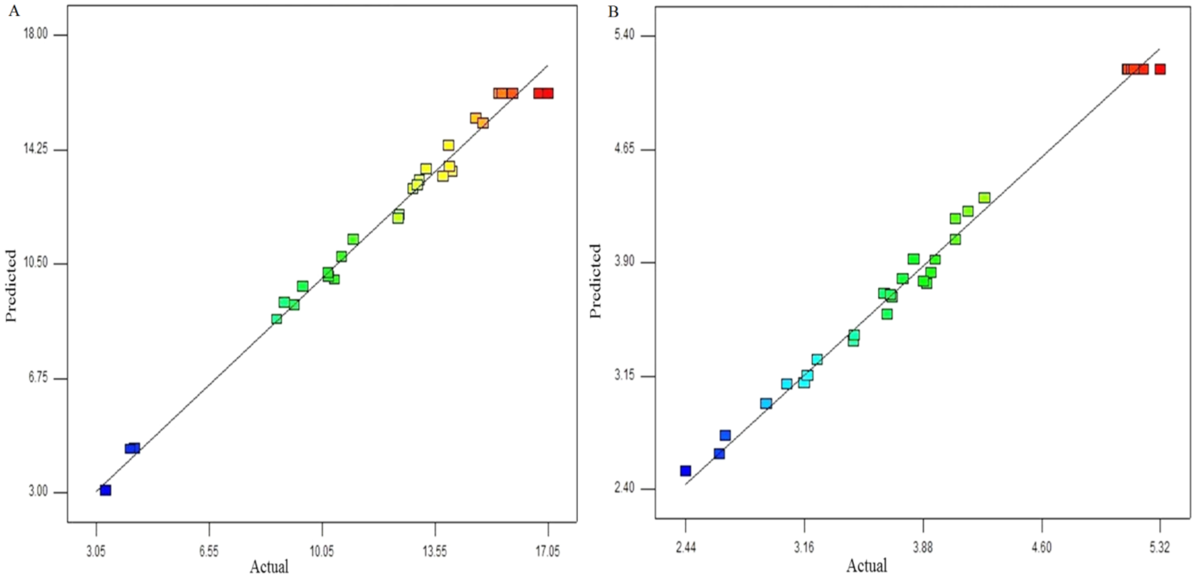

Fig. S2 Neural Network Architecture

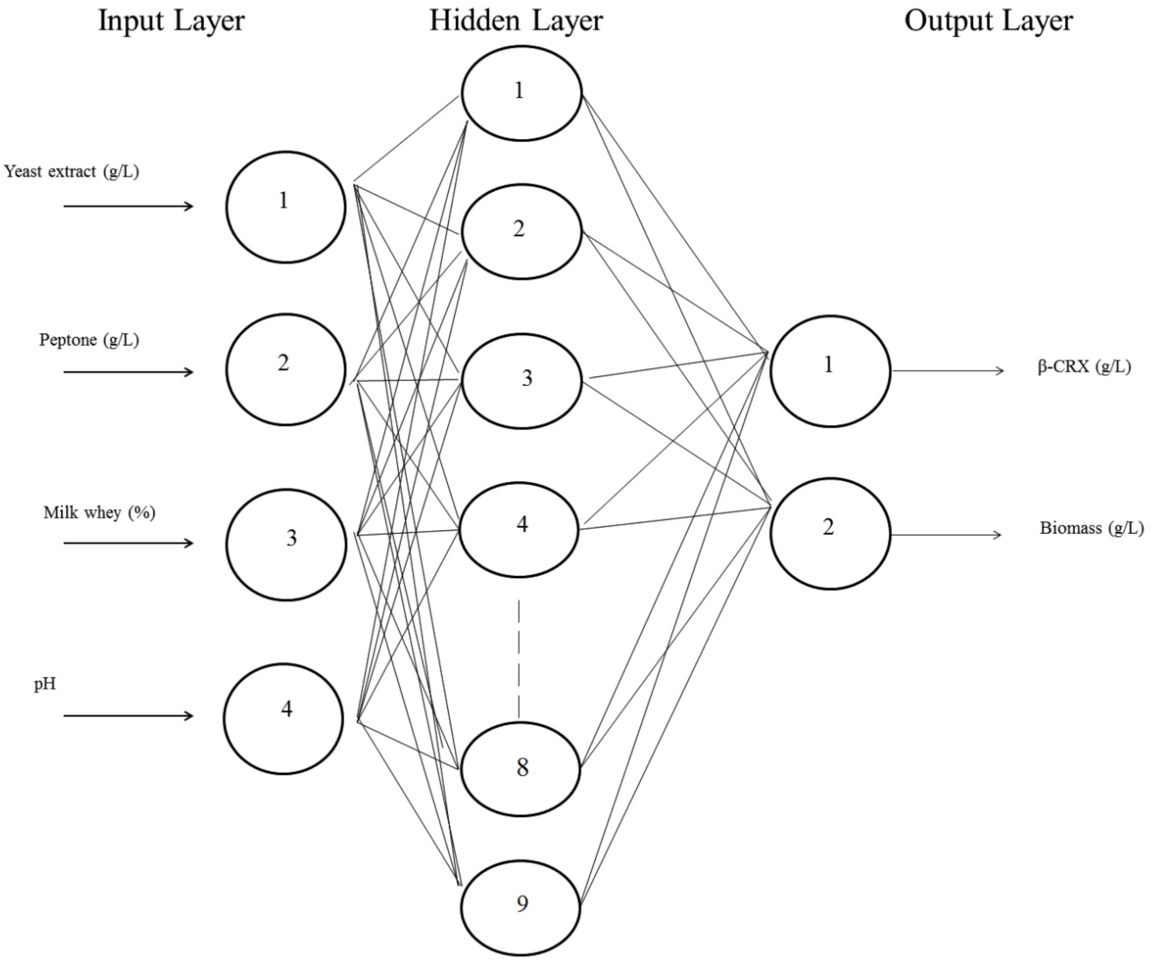

Fig. S3 ANN performance plot

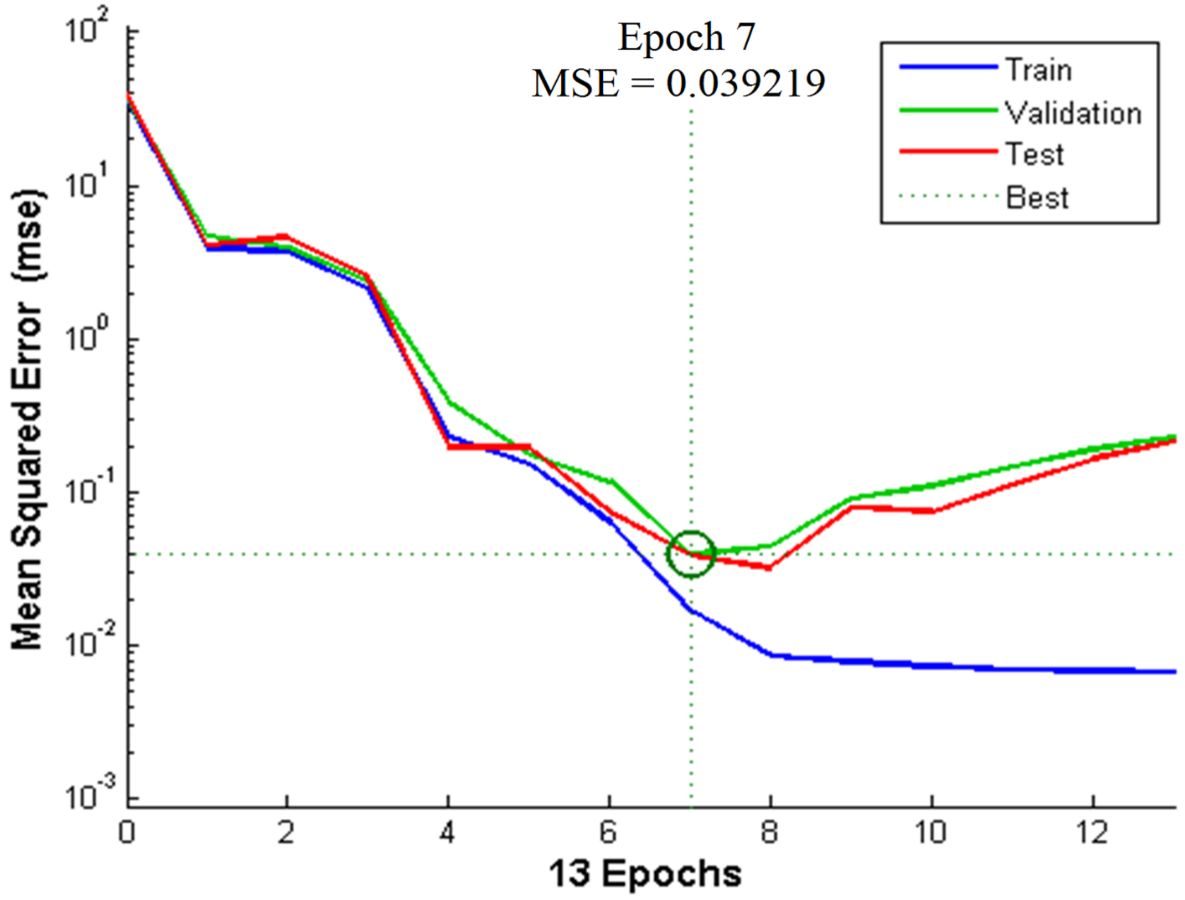

Fig. S4 Error histogram for the ANN predictive model

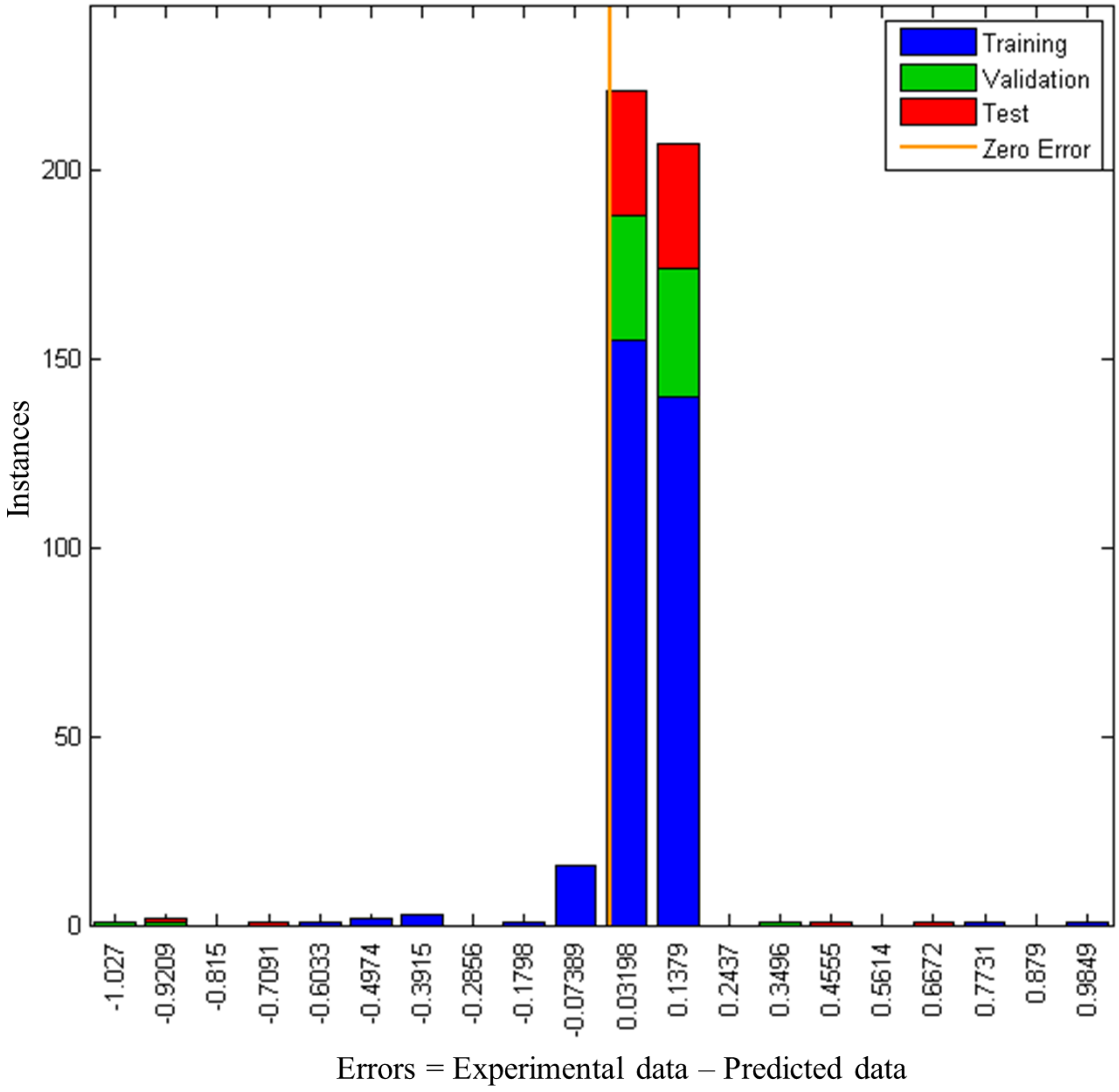

Table S1 Coded values of independent variables

Factors	Variables	Units	Range of levels				
			-2	-1	0	+1	+2
A	Yeast extract	g/L	0.0	5.0	10.0	15.0	20.0
B	Peptone	g/L	0.0	2.5	5.0	7.5	10.0
C	Cheese whey	% (v/v)	0.0	5.0	10.0	15.0	20.0
D	Initial pH	Unit	6.25	7.0	7.75	8.5	9.25

Table S2 Substrate inhibition kinetic models used in the study

Sl. No.	Name of model	Equation	References
01	Monod	$\mu = \frac{\mu_{max} S}{S + K_S}$	Monod 1949
02	Moser	$\mu = \frac{\mu_{max} S^n}{K_S + S^n}$, n > 0	Moser 1985
03	Aiba	$\mu = \frac{\mu_{max} S}{S + K_S} exp^{(\frac{-S}{K_i})}$	Aiba *et al.* 1968
04	Andrews and Noack	$\mu = \frac{\mu_{max} S}{S + K_S + (\frac{S^2}{K_i})}$	Andrews 1968
05	Han and Levenspiel	$\mu = \mu_{max}(1 - \frac{S}{S_m})^n (\frac{S}{S + K_S(1 - (\frac{S}{S_m}))^m})$	Han and Levenspiel 1988
06	Luong substrate inhibition	$\mu = \frac{\mu_{max} S}{S + K_S}[1 - \frac{S}{S_m}]^m$	Luong 1987
07	Luong product inhibition	$\mu = \frac{\mu_{max} S}{S + K_S}[1 - (\frac{S}{S_m})^n]$	Luong 1985
08	Tiessier-type substrate inhibition	$\mu = \mu_{max}[\exp\left(\frac{-S}{K_i}\right) - \exp\left(\frac{-S}{K_S}\right)]$	Edward 1970
09	Edward	$\mu = \mu_{max} \frac{S}{S + K_S + (\frac{S^2}{K_i})(1 + \frac{S}{K_S})}$	Edward 1970
10	Webb	$\mu = \frac{\mu_{max} S \left(1 + \left(\frac{S}{K_i}\right)\right)}{\left(K_S + S + \left(\frac{S^2}{K_i}\right)\right)}$	Edward 1970
11	Yano and Koga	$\mu = \mu_{max} \frac{S}{K_S + S + \frac{S^2}{K_1} + \frac{S^3}{K_2}}$	Yano and Koga 1969
12	Tseng and Wayman	$\mu = \left(\mu_{max}\left(\frac{S}{K_S + S}\right)\right) - (K_i(S - S_m))\ S > S_m$	Tseng and Wayman 1975

Table S3 Estimated values of kinetic parameters as obtained by the kinetic modelling at different initial cheese whey concentrations

Sl. No.	Name of model	μ_{max} (h^{-1})	K_S	K_i	K_1	K_2	S_m	m	n	R^2	σ X 10^4	$SS\ X\ 10^4$	$Sy.x\ X\ 10^4$ (h^{-1})	MSE (% $X\ 10^4$
01	Monod	0.1731	5.292	----	---	----	----	---	---	0.8475	23.1200	29.57	222.0026	369.6394
02	Moser	0.1340	62.650	----	---	----	----	---	2.8550	0.9143	25.0383	16.61	182.2558	207.6072
03	Aiba	0.5371	20.590	27.3100	----	---	----	----	----	0.9133	23.1541	16.81	167.3674	210.0891
04	Andrews and Noack	0.1731	5.292	$5.025\ X\ 10^{16}$	----	---	----	----	----	0.8475	23.1249	29.57	243.1917	369.6399
05	Han and Levenspiel	0.2385	22.06	----	---	----	27.40	3.8680	0.5576	0.9982	22.4991	0.3816	30.8900	47.70
06	Luong substrate inhibition	0.5781	27.060	----	---	----	23.51	0.3776	----	0.9688	22.7594	6.057	100.4703	75.7075
07	Luong product inhibition	0.4531	23.700	----	---	----	25.92	----	3.1800	0.9801	22.6658	3.853	87.7814	48.1625
08	Tiessier-type substrate inhibition	0.3527	9.251	26.74	----	---	----	----	----	0.9224	23.0709	15.05	158.3903	188.1561
09	Edward	0.5221	23.980	18.410	----	---	----	----	----	0.9415	22.8419	11.35	137.5186	141.8352
10	Webb	0.1408	$1.474\ X\ 10^4$	0.001217	----	---	----	----	----	0.9018	24.2226	19.03	195.1094	237.9231
11	Yano and Koga	0.6381	32.950	----	$9.775\ X\ 10^{17}$	151.5	----	----	----	0.9677	22.7741	6.268	111.9652	78.3513
12	Tseng and Wayman	0.1732	5.312	$6.721\ X\ 10^{-6}$	----	----	26.90	----	----	0.8475	23.1033	29.57	243.1903	369.6396
